# Visualization of Fluoride Ions In Vivo Using a Gadolinium(III)-Coumarin Complex-Based Fluorescence/MRI Dual-Modal Probe

**DOI:** 10.3390/s16122165

**Published:** 2016-12-16

**Authors:** Yue Wang, Renfeng Song, Huan Feng, Ke Guo, Qingtao Meng, Haijun Chi, Run Zhang, Zhiqiang Zhang

**Affiliations:** 1 School of Chemical Engineering, University of Science and Technology Liaoning, Anshan 114051, China; Wangyue9088@163.com (Y.W.); FengHuan960421@163.com (H.F.); chi_haijun@163.com (H.C.); henrry1964@163.com (Z.Z.); 2 Ansteel Mining Engineering Corporation, Anshan 114002, China; srfandwl@163.com (R.S.); asgk2006@sina.com (K.G.); 3 Australian Institute for Bioengineering and Nanotechnology, The University of Queensland, Brisbane 4072, Australia

**Keywords:** gadolinium(III) complex, dual-modal, detection, fluorescence/MRI, fluoride ion

## Abstract

A new Gadolinium(III)–coumarin complex, DO3A-Gd-**CA**, was designed and prepared as a dual-modal probe for simultaneous fluorescence and relaxivity responses to fluoride ions (F^−^) in aqueous media and mice. DO3A-Gd-**CA** was designed by using Gd(III) center as an MRI signal output unit and fluoride binding site, and the 4-(diethylamino)-coumarin-3-carboxylic acid (**CA**) as a fluorescence reporter. Upon the addition of fluoride ions to the solution of DO3A-Gd-**CA**, the liberation of the coordinated **CA** ligand led to a 5.7-fold fluorescence enhancement and a 75% increase in the longitudinal relaxivity (*r*_1_). The fluorescent detection limit for fluoride ions was determined to be 8 μM based on a 3*σ*/*slope*. The desirable features of the proposed DO3A-Gd-**CA**, such as high sensitivity and specificity, reliability at physiological pH and low cytotoxicity enable its application in visualization of fluoride ion in mice. The successful in vivo imaging indicates that DO3A-Gd-**CA** could be potentially used in biomedical diagnosis fields.

## 1. Introduction

Detection and diagnosis of diseases at early stage with a high level of accuracy is the key factor to be considered in biomedical and clinical researches on the treatment of diseases [1,2]. The disease biomarkers are often detected at all stages during diseases diagnostic and treatment, while such detection remains a challenge currently [3,4]. Molecular imaging enables visualization of disease biomarkers and their metabolisms in living systems in real time at various levels from molecules and single cells, to tissues, and organs, which allows detection and differential diagnosis of diseases [5,6]. Over the past decades, a variety of molecular imaging techniques, such as positron emission tomography (PET), X-ray computed tomography (CT), magnetic resonance imaging (MRI) and fluorescence imaging (FI), have been widely used in clinical diagnostics, biomedical research and molecular imaging fields [7,8,9,10]. Each method has its inherent advantages and considerations. For instance, a magnetic resonance imaging (MRI) technique is characterized by superb spatial resolution but low sensitivity and requires a high concentration of the contrast agent [11]. Fluorescence imaging, on the other hand, has much higher sensitivity and the potential for real-time imaging, but with limited penetration depth of optical photonics, which restricts their application in the detection of biomarkers in deep tissues [12].These limitations could be addressed by the dual-modal or multi-modal imaging techniques proposed recently [13,14,15]. A fluorescence/MRI dual-modal probe fabricated by integrating a fluorescent unit with a contrast agent could thus be used for imaging of biomarkers in vivo. With these dual-modal probes, detailed information on biomarkers in cells and organs within live bodies could be observed simultaneously employing fluorescence imaging and MRI techniques [16].Therefore, molecular probes, defined as a class of molecules that bind selectively to the analyte of interest with concomitant change in one or more properties of the system, such as fluorescence color [17,18,19], redox potential and magnetic resonance [20], plays fundamental roles in advanced imaging techniques [21]. In recent years, numerous efforts have been made for the development of molecular probes for the monitoring of biomarkers in biological systems [22]. Nevertheless, to the best of our knowledge, anions fluorescence/MRI dual-modal probes based on Gd(III) complex have been scarcely reported [23].

Due to the important roles of anions played in live organisms, environmental samples, and in the broad applications (e.g., catalysis, etc.), the development of molecular probes for the selective and sensitive sensing and quantification of anions of interest have attracted considerable interest [24]. Among various important anions in biological systems, fluoride ion, being the smallest and most electronegative anion, has particularly attracted attention due to its profound effects on human health. It is well known that intake of acute amount of fluoride ions is beneficial to the treatment of osteoporosis and dental health [25]. On the other hand, excessive intake of fluoride ions may induce various diseases, such as gastrointestinal dysfunction, dental fluorosis, bone fluorosis, and so on [26,27]. Accordingly, the development of molecule probes for the selective and sensitive detection of fluoride ion in biological systems is urgently needed.

**Scheme 1:**
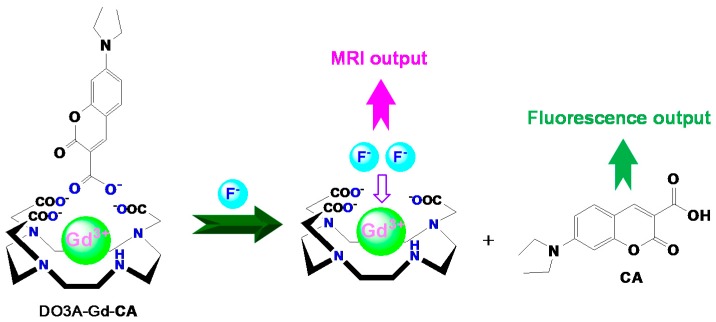
Proposed dual-modal responses mechanism of DO3A-Gd-**CA** towards fluoride ion.

In recent years, lanthanide (Ln) complexes have been reported to be an excellent candidate for the development of the responsive optical probes owing to their unique photophysical properties [28,29,30]. By virtue of the extremely high Ln–fluoride affinity, the coordinated molecules or fluorophores of Ln complexes can be displaced by fluoride ions. As the result of this interaction, the liberation of the coordinated ligands may lead to the changes in several observable signals, such as colour [31], fluorescence and MR [32,33]. Due to their high magnetic moment and symmetric electronic ground state, gadolinium(III) complexes have been frequently chosen as MRI contrast agents [34]. As a consequence of the fluoride ions-induced displacement, the relaxivity of Gd(III) complexes thus could be finely tuned by changing in *q* (number of coordinated water molecular) upon the addition of targeted fluoride ions in aqueous solution (Scheme 1) [35,36,37,38,39,40,41,42,43].

## 2. Materials and Methods

### 2.1. Materials and Instruments

4-Diethylaminosalicylaldehyde, diethylmalonate, piperidine and tetrabutylammonium salts of F^−^, Cl^−^, Br^−^, I^−^, HSO_4_^−^, NO_3_^−^, OH^−^, AcO^−^, PO_4_^3−^ and H_2_PO_4_^−^ were purchased from Sinopharm Chemical Reagent Co., Ltd. (Shanghai, China). GdCl_3_, 1,4,7,10-Tetraazacyclododecane (Cyclen), tert-Butyl bromoacetate and trifluoroacetic acid were purchased from Alfa Aesar.

^1^H-NMR and ^13^C-NMR spectra were recorded with an AVANCE600MHZ spectrometer (BRUKER) with chemical shifts reported as ppm (in DMSO, TMS as internal standard). API-ES mass spectra were recorded on an Agilent 6530QTOF spectrometer. Fluorescence spectra were determined with LS 55 luminescence spectrometer (Perkin Elmer, Waltham, MA, USA). The absorption spectra were measured with a Lambda 900 UV/VIS/NIR spectrophotometer (Perkin Elmer, Waltham, MA, USA). Longitudinal relaxivity (*r*_1_) and MR imaging were performed on an MesoMR23-060H-I Analyst Analyzing & Imaging system (Shanghai Niumag Corp., Shanghai, China) using a 0.5 T magnet, point resolution = 256 × 128 mm, section thickness = 1 mm, TE = 18.2 ms, TR = 400 ms, and number of acquisitions = 4.

### 2.2. Synthesis and Characterization of 4-(Diethylamino)-coumarin-3-carboxylic acid (**CA**)

4-(diethylamino)-coumarin-3-carboxylic acid (**CA**) was synthesized according to the literature procedurein 78% yield [44]. ^1^H-NMR (DMSO-*d*, 600 MHz) δ (ppm): 12.52 (s, H), 8.59 (s, 1H), 7.64 (d, *J* = 12.0 Hz, 2H), 6.79 (d, *J* = 12.0 Hz, 2H), 6.57 (s, 1H), 3.49 (m, *J* = 9.0 Hz, 4H), 1.14 (t, *J* = 9.0 Hz, 6H). ^13^C-NMR (DMSO-*d*, 150 MHz) δ (ppm): 165.0, 160.0, 158.4, 153.4, 149.9, 132.3, 110.5, 107.8, 107.7, 96.4, 44.9, 12.8. ESI-HRMS (positive mode, *m*/*z*) Calcd for C_14_H_15_NO_4_: 261.1001 [**CA** − H]^+^; Found: 260.0995.

### 2.3. Synthesis and Characterization of DO3A-Gd

1,4,7-Tris(carboxymethyl)-1,4,7,10-tetraazacyclododecane (DO3A) and DO3A-Gd were synthesized according to the literature procedure [45,46]. ESI-HRMS (positive mode, *m*/*z*) of DO3A, Calcd for C_14_H_27_N_4_O_6_: 347.1931: [DO3A + H]^+^; Found: 347.1936; ESI-HRMS (positive mode, *m*/*z*) of DO3A-Gd, Calcd for C_14_H_22_GdN_4_O_6_: 500.0802 [DO3A-Gd]^−^; Found: 500.0781.

### 2.4. Synthesis and Characterization of DO3A-Gd-**CA**

DO3A-Gd-**CA** was prepared by addition of 1.0 equiv. of [DO3A-Gd]Na to **CA** (10 μM) solution in CH_3_CN-H_2_O (9:1, *v*/*v*, pH = 7.4). ESI-HRMS (positive mode, *m*/*z*) of [DO3A-Gd-**CA**]**^−^**, Calcd for C_28_H_37_GdN_5_O_10_: 761.1803, Found: 761.1778.

### 2.5. General Procedures of Spectroscopic Detection

Stock solutions of **CA** (1 mM) were prepared in CH_3_CN. Before spectroscopic measurements, the solution was freshly prepared by diluting the high concentration of stock solution to corresponding solution (10 μM, CH_3_CN:H_2_O = 9:1, pH = 7.4). DO3A-Gd-**CA** stock solution for anions sensing was prepared in situ by addition of 1.0 equiv. of [DO3A-Gd]Na to **CA** (10 μM) solution in CH_3_CN-H_2_O (9:1, *v*/*v*, pH = 7.4). Each time, a 3 mL solution of probe was filled in a quartz cell of 1 cm optical path length, and different stock solutions of cations were added into the quartz cell gradually by using a micro-syringe. Excitation wavelength for **CA** was 408 nm.

### 2.6. Association Constant Calculation

The Benesi-Hildebrand equation was used as shown below [47].

$$\frac{1}{F_{0}-F}=\frac{1}{K_{a}(F_{0}-F_{\min})[\mathrm{DO}3A\mathrm{-Gd}]}+\frac{1}{F_{0}-F_{\min}}$$

where *F* and *F*_0_ represent the fluorescence emission at 460 nm of **CA** in the presence and absence of [DO3A-Gd]^−^, respectively, *F*_min_ is the saturated fluorescence intensity.

### 2.7. MRI Experiment

Male 8-week-old mice were purchased from the Animal Experiment Center of Dalian Medical University. All live mice experiments were performed in compliance with the relevant laws and institutional guidelines.

All MR relaxivity measurements were performed on a MesoMR23-060H-I Analyzing & Imaging system (Shanghai Niumag Corp.). The specific relaxivity values of *r*_1_ were calculated through the curve fitting of 1/T_1_ (s^−1^) vs. the concentration of DO3A-Gd-**CA** (0.2 mM). T_1_-weighted MR images were acquired using a multi-slice gradient echo sequence.

## 3. Results

### 3.1. Synthesis and Photophysical Characterization of DO3A-Gd-**CA** in Solution

**Figure 1:**
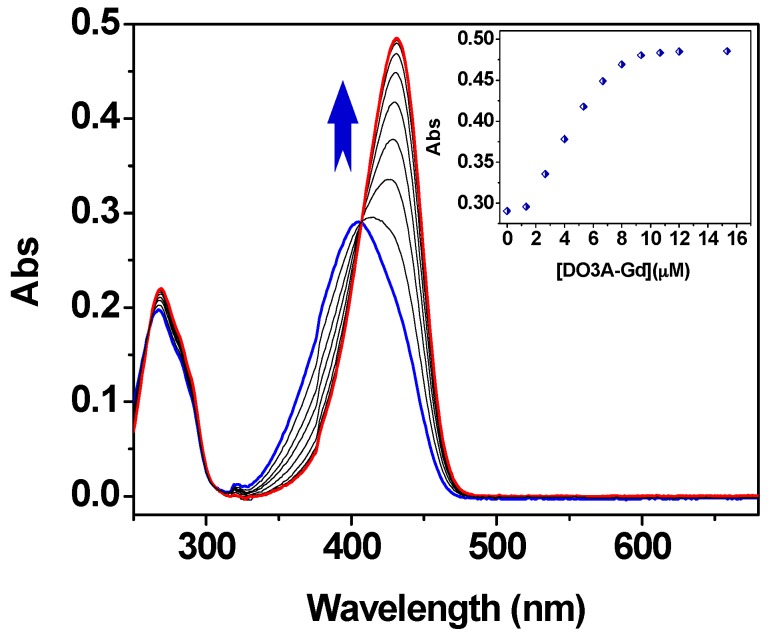
UV-Vis absorption spectra of **CA** (10 μM) in the presence of increasing amount of DO3A-Gd (0–15.0 μM) in CH_3_CN-H_2_O (9:1, *v*/*v*, pH = 7.4). Insert: The maximum absorbance band of **CA** at 430 nm against the concentrations of DO3A-Gd.

The formation of DO3A-Gd-**CA** was investigated by recording the changes in UV-Vis absorption and fluorescence spectra. As shown in Figure 1, consistent with the free coumarin derivative, **CA** displayed a major absorption maximum at 402 nm in aqueous medium (CH_3_CN:H_2_O = 9:1, pH = 7.4) [48]. In the presence of an increasing amount of [DO3A-Gd]^−^ (0–15 µM), an obvious bathochromic-shift in the absorption band from 402 nm to 430 nm and an increase in the absorption at 430 nm were noted, which could be assigned to the complexation of **CA** with Gd(III) center.

**Figure 2:**
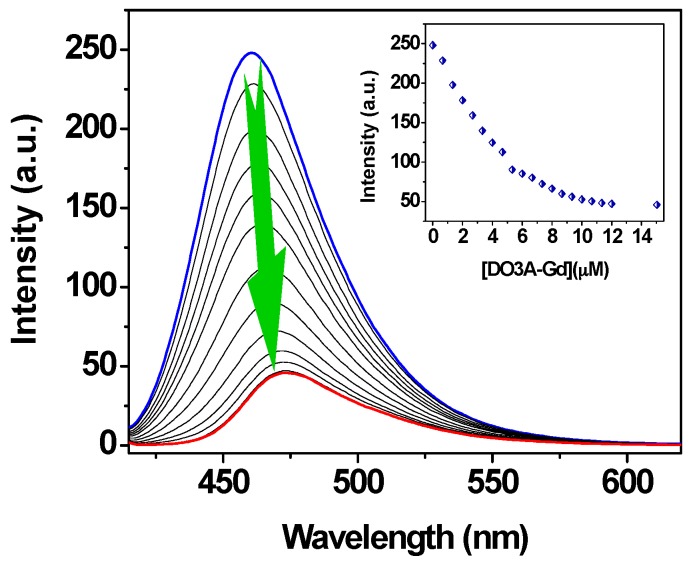
Fluorescence spectra of **CA** (10 μM) in CH_3_CN-H_2_O (9:1, *v*/*v*, pH = 7.4) in the presence of different amounts of DO3A-Gd (0–15.0 μM). Insert: Fluorescence intensities of **CA** at 460 nm as a function of the concentration of DO3A-Gd. Excitation was performed at 408 nm.

The complexation of **CA** with [DO3A-Gd]^−^ was further studied by the analysis of fluorescence titration. **CA** displayed strong fluorescence at 460 nm in CH_3_CN-H_2_O (9:1, *v*/*v*, pH = 7.4). As expected, the fluorescence intensity of **CA** decreased gradually with a 12 nm bathochromic-shift in the emission band in the presence of an incremental addition of [DO3A-Gd]^−^. The fluorescence intensity reached a constant value when 12 µM fluoride ion was added (Figure 2, insert). The decrease of fluorescence intensity (more than 81.5% fluorescence quenching) and bathochromic-shift of emission wavelength suggested the formation of the complex DO3A-Gd-**CA** [49,50]. Based on the 1:1 binding mode, the association constant (*K*_a_) was evaluated to be 4.3 × 10^3^ M^−1^ using the Benesi–Hildebrand method (Figure S6) [51]. Furthermore, no obvious changes of the fluorescent intensities within 13 h, indicating that DO3A-Gd-**CA** is reliable under the test condition (Figure S7). The proposed structure of DO3A-Gd-**CA** was further confirmed by ESI-HRMS. As shown in Figure S8, analysis of the solution of DO3A-Gd-**CA** revealed the presence of a peak assigned to [DO3A-Gd-**CA**]^−^ at *m*/*z* = 761.1778, indicating the formation of the expected 1:1 stoichiometry complex between DO3A-Gd and **CA**.

### 3.2. Spectroscopic Studies of DO3A-Gd-**CA** towards Fluoride Ion in Aqueous Medium

**Figure 3:**
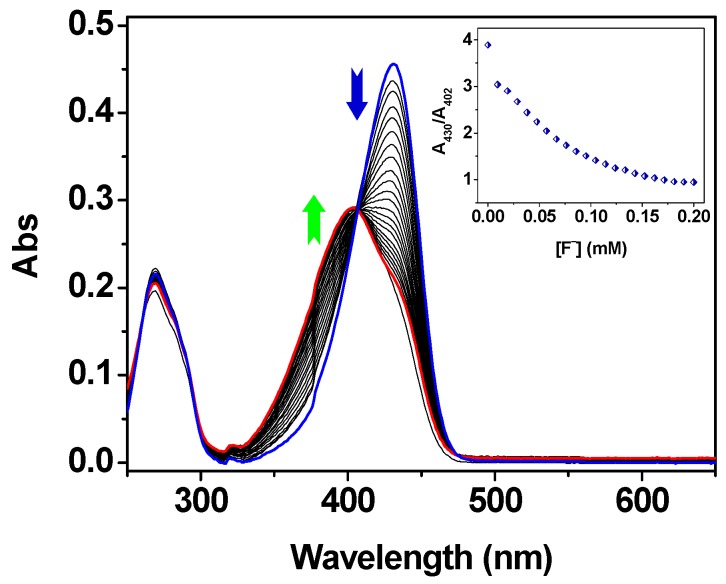
UV-Vis absorption spectra of DO3A-Gd-**CA** (10 μM) in the presence of increasing concentrations of fluoride ions (0–200 μM) in CH_3_CN-H_2_O (9:1, *v*/*v*, pH = 7.4). Insert: Ratiometric changes of absorbance band of **CA** (A_430_/A_402_) as a function of fluoride ions concentrations.

**Figure 4:**
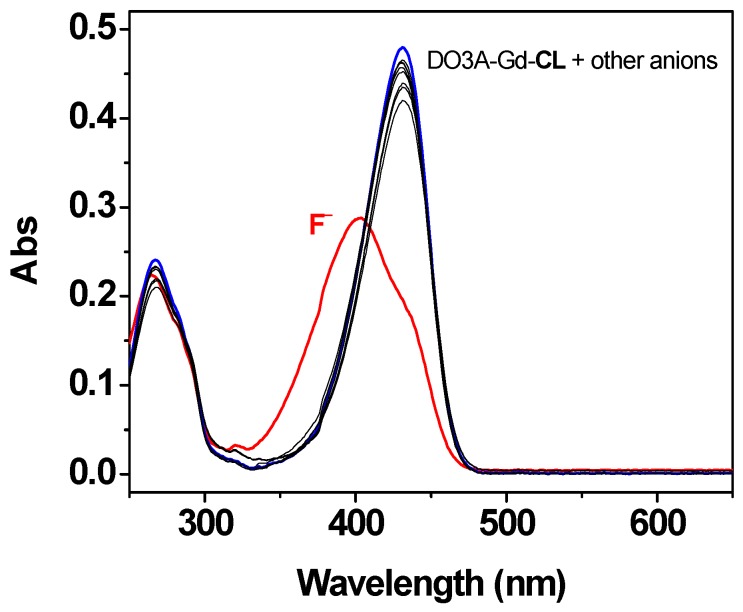
UV-Vis absorption spectra of DO3A-Gd-**CA** (10 μM) in the presence of various anions (200 μM) in CH_3_CN-H_2_O (9:1, *v*/*v*, pH = 7.4): H_2_PO_4_^−^ (0.2 mM), PO_4_^3−^ (1 mM), HSO_4_^−^ (0.2 mM), Br^−^(0.2 mM), I^−^ (0.2 mM), NO_3_^−^ (0.2 mM), OH^−^ (0.2 mM), AcO^−^ (0.2 mM), Cl^−^ (100 mM), F^−^ (0.2 mM).

By virtue of the high affinity of gadolinium(III) center and fluoride ions, fluoride is expected to replace the coordinated **CA** ligand [52], resulting the significant changes in UV-Vis and fluorescence spectra of **CA**, by which fluorescence detection of fluoride ion could be achieved. The stock solution of DO3A-Gd-**CA** for anion sensing was prepared in situ by the addition of 1.0 equiv. of [DO3A-Gd]^−^ to **CA** solution (10 μM) in CH_3_CN-H_2_O (9:1, *v*/*v*, pH = 7.4). The displacement approach was firstly investigated by UV-Vis spectra titration analysis. The major absorption band of DO3A-Gd-**CA** (10 μM) at 430 nm decreased and the absorption peak assigned to free **CA** at 402 nm emerged by the addition of 0–200 μM tetrabutylammonium fluoride (n-Bu_4_NF) to the DO3A-Gd-**CA** solution (Figure 3). The final absorption spectrum was in agreement with the native **CA** in identical conditions (Figure S9a), which corroborated the results of the decomplexing of DO3A-Gd-**CA** in the presence of fluoride ion. The changes of absorption spectra ofDO3A-Gd-**CA** in the presence of other physiologically and environmentally important anions, such as Br^−^, I^−^, AcO^−^, HSO_4_^−^, NO_3_^−^, OH^−^ and H_2_PO_4_^−^ were then evaluated by UV-Vis analysis. As illustrated in Figure 4, obvious changes in UV-Vis spectra were obtained upon the addition of fluoride ions, instead of other anions even at high concentrations (concentration of Cl^−^, PO_4_^3−^ are 100 mM and 1 mM, respectively). This result indicates that DO3A-Gd-**CA** is specific toward fluoride ions over other competitive anions.

**Figure 5:**
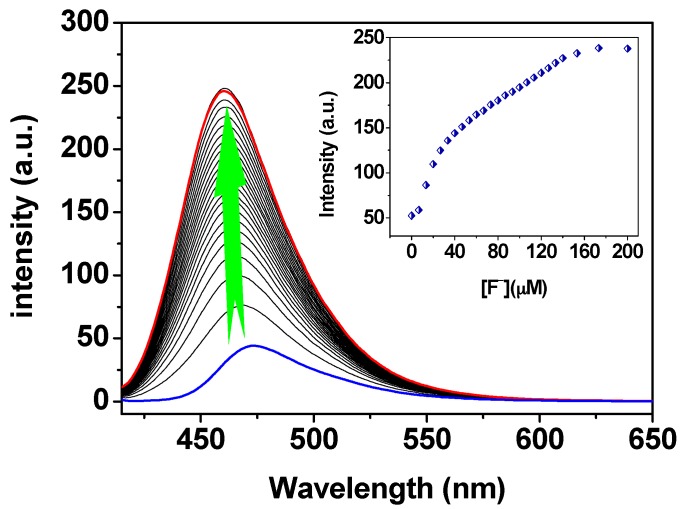
Fluorescence spectra of DO3A-Gd-**CA** (10 μM) in the presence of different concentrations of fluoride ion (0–200 μM). Insert: Fluorescence intensities of DO3A-Gd-**CA** (10 μM) at 460 nm as a function of fluoride ions concentration (0–200 μM). Excitation was performed at 408 nm.

**Figure 6:**
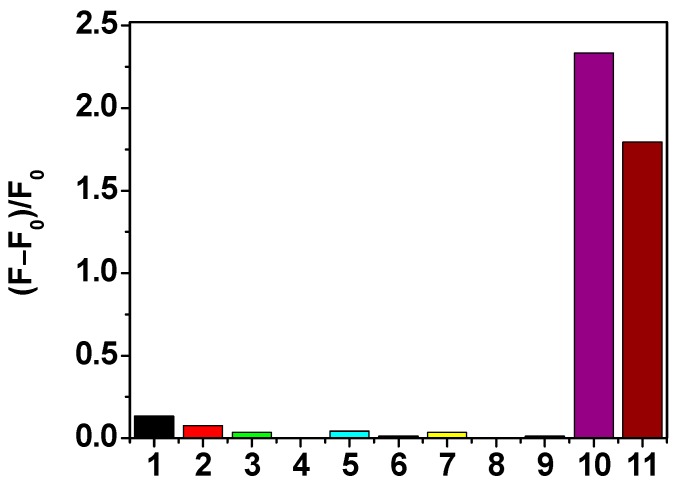
Changes in fluorescence intensity of DO3A-Gd-**CA** (10 μM) toward fluoride ion in the presence of various competing anions in CH_3_CN-H_2_O (9:1, *v*/*v*, pH = 7.4): 1. H_2_PO_4_^−^ (0.2 mM), 2. PO_4_^3−^ (1 mM), 3. HSO_4_^−^ (0.2 mM), 4. Br^−^ (0.2 mM), 5. I^−^ (0.2 mM), 6. NO_3_^−^ (0.2 mM), 7. OH^−^ (0.2 mM), 8. AcO^−^ (0.2 mM), 9. Cl^−^ (100 mM), 10. F^−^ (0.2 mM), and 11. All anions mixed. The intensities were recorded at 460 nm, excitation at 408 nm.

To evaluate the capability of DO3A-Gd-**CA** as the fluorescence probe for fluoride ions, emission spectra of DO3A-Gd-**CA** were then investigated. As shown in Figure 5, DO3A-Gd-**CA** presented a weak fluorescence emission. Upon the addition of fluoride ion, the maximum emission wavelength of DO3A-Gd-**CA** at 472 nm was gradually shifted to 460 nm, accompanied by an increase in fluorescence intensity. When 200 μM fluoride ion was added, the fluorescence intensity increased to a maximum value. The intensity and overall pattern of the emission spectrum closely match those of native **CA** state, indicating the liberation of coordinated **CA** ligand (Figure S9b). Job’s plots of the fluorescence emission variation at 460 nm against the mole fraction of fluoride ions clearly showed the inflection point at 0.67 (Figure S10), which supported that DO3A-Gd-**CA** binding fluoride ion with 1:2 binding stoichiometry. The fluoride-induced replacement process was further verified by HRMS. Upon the addition of fluoride ions into the aqueous solution of DO3A-Gd-**CA**, the peak of [DO3A-Gd-**CA**]^−^ at *m*/*z* = 761.1778 disappeared, and a new peak at *m*/*z* = 260.0930 assigned to [**CA**–H^+^]^−^ was emerged, indicating that the binding between fluoride ions with Gd(III) centers led to the release of **CA** (Figure S11). To evaluate the selectivity of DO3A-Gd-**CA** toward fluoride ion against other anions, the changes in fluorescence of DO3A-Gd-**CA** were examined in the presence of 0.2 mM various anions (Br^−^, I^−^, H_2_PO_4_^−^, HSO_4_^−^, AcO^−^, NO_3_^−^, OH^−^, AcO^−^) and 100 mM Cl^−^, 1 mM PO_4_^3−^. The maximum emission of DO3A-Gd-**CA** significantly increased in the presence of fluoride ions exclusively, whereas other anions did not induce any detectable fluorescence enhancement (Figure 6). To further verify the excellent selectivity for fluoride ions, the competitive experiments were conducted by the addition of 0.2 mM fluoride ion to the mixture of DO3A-Gd-**CA** and diverse coexisting anions. As shown in Figure 6, negligible effects on the detection of fluoride ions were found in the presence of various completive anions, indicating DO3A-Gd-**CA** is a fluorescence probe specific for fluoride ion detection [53]. The excellent selectivity to fluoride ions is partly due to the highest affinity to gadolinium(III) center compared to other anionic analytes [54]. The relative fluorescence intensity of DO3A-Gd-**CA** is linearly proportional to fluoride ions concentration of 0–30 μM (Figure S12), and the detection limit was calculated to be 8 μM based on a 3*σ*/slope under experimental conditions, which is low enough for fluoride ion sensing in aqueous medium and biological systems [55].

### 3.3. MRI Responses of DO3A-Gd-**CA** towards Anions in Aqueous Medium and In Vivo

**Figure 7:**
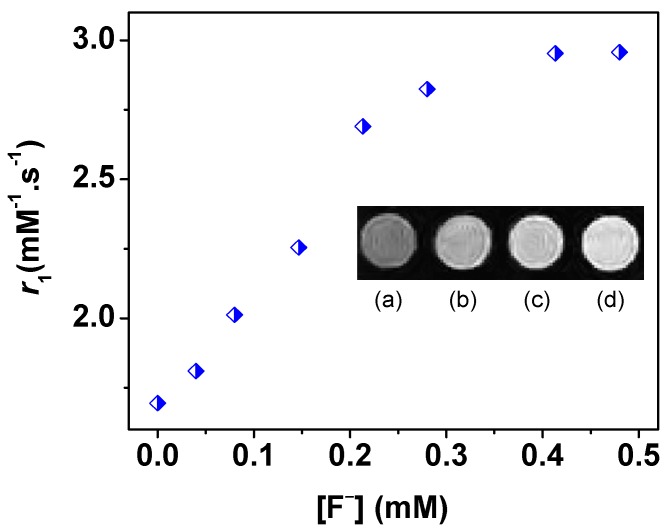
Changes in the longitudinal relaxivity (*r*_1_) of DO3A-Gd-**CA** (0.2 mM) as a function of the fluoride ion concentration (0–0.5 mM) in CH_3_CN-H_2_O (9:1, *v*/*v*, pH = 7.4). Insert: T_1_-weighted MR images of DO3A-Gd-**CA** recorded versus different concentrations of fluoride ion: (**a**) 0 mM; (**b**) 0.1 mM; (**c**) 0.3 mM; (**d**) 0.5 mM.

The longitudinal relaxivity (*r*_1_) of DO3A-Gd-**CA** towards fluoride ions was further investigated by a MesoMR23-060H-I Analyzing & Imaging system at 25 °C. As shown in Figure 7, DO3A-Gd-**CA** (0.2 mM) exhibited relatively low longitudinal relaxivity (*r*_1_ = 1.67 mM^−1^∙s^−1^), suggesting that no inner-sphere water molecule was bonded to Gd(III) ion arising from the complexation of EDTA-Gd with **CA** (*q* = 0) [56]. Upon the addition of fluoride ion into the aqueous solution containing EDTA-Gd-CA (0.2 mM), the relaxivity increased to 2.957 mM^−1^·s^−1^ (Figure 7), which closely matched the native EDTA-Gd state (Figure S13). The result demonstrated the replacement of coordination **CA** in the presence of fluoride ions. In addition, the corresponding T_1_-weighted images of DO3A-Gd-**CA** (0.2 mM) presented a continuous increase in spot brightness with increasing fluoride ion concentrations (Figure 7 insert). The increase in longitudinal relaxivity (*r*_1_) demonstrated the applicability of DO3A-Gd-**CA** as an efficient T_1_ MRI probe.

**Figure 8:**
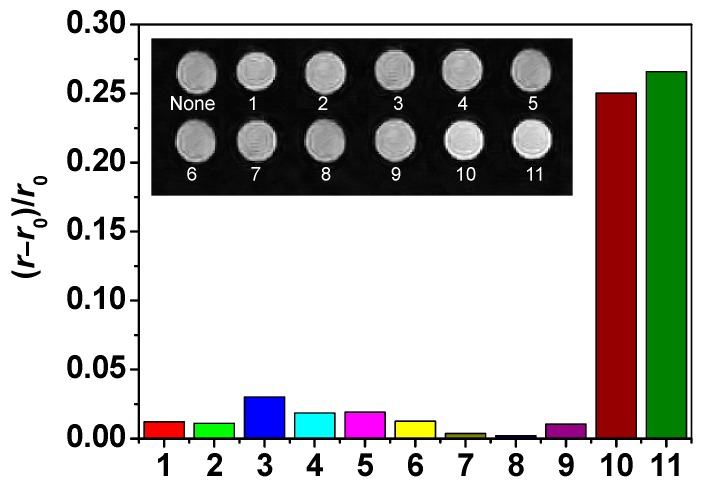
Longitudinal relaxivity (*r*_1_) responses of DO3A-Gd-**CA** (0.2 mM) to various anions of interest (0.5 mM) in CH_3_CN-H_2_O (9:1, *v*/*v*, pH = 7.4). Insert: T_1_-weighted MR images (T_1_ measurements at a proton frequency of 20 MHz) of DO3A-Gd-**CA** (0.2 mM ) in the presence of (1) Br^−^ (0.5 mM); (2) I^−^ (0.5 mM); (3) Cl^−^ (100 mM); (4) PO_4_^3−^ (1.0 mM); (5) H_2_PO_4_^−^ (0.5 mM); (6) HSO_4_^−^ (0.5 mM); (7) NO_3_^−^ (0.5 mM); (8) OH^−^ (0.5 mM); (9) AcO^−^ (0.5 mM); (10) F^−^ (0.5 mM); and (11) All anions mixture.

The changes in relaxivity of DO3A-Gd-**CA** were fluoride-specific over other physiologically important anions including Br^−^, I^−^, H_2_PO_4_^−^, HSO_4_^−^, AcO^−^, NO_3_^−^, OH^−^, AcO^−^, Cl^−^ (100 mM) and PO_4_^3−^ (1 mM) (Figure 8). In addition, a distinctly increased imaging intensity in the presence of 0.5 mM fluoride ions was obtained, whereas no obvious differences were observed upon addition of other competitive anions (Figure 8, insert). In order to investigate the feasibility of DO3A-Gd-**CA** for the in vivo magnetic resonance imaging (MRI), the long-term cellular toxicity of DO3A-Gd-**CA** was evaluated by means of a MTT assay [57,58]. The MDA-MB-231 cell viabilities remained more than 80% at a high concentration of 200 µM even with an incubation time of 24 h, demonstrating the low cytotoxicity of DO3A-Gd-**CA** (Figure S14). In addition, the suitable pH range for DO3A-Gd-**CA** was evaluated to be 3.0–11.0, suggested that the probe is suitable for application under physiological conditions (Figure S15).

**Figure 9:**
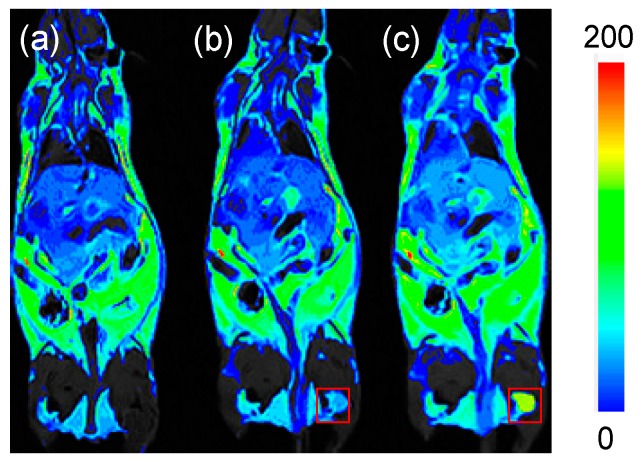
T_1_-weighted MR pseudocolor images of living white mice injection of: (**a**) Control group; (**b**) DO3A-Gd-**CA** (0.2 mL, 0.2 mM) only; (**c**) DO3A-Gd-**CA** (0.2 mL, 0.2 mM) + 0.5 mM fluoride ions.

Then, in vivo MRI experiments were studied using a 0.5 T MRI instrument. As shown in Figure 9b, after the injection of DO3A-Gd-**CA** (0.2 mM), a 40.1% contrast enhancement at the site of injection was observed compared with the baseline of the pre-injection image (Figure 9a). Further injection of fluoride ions (0.2 mL, 0.5 mM) into the hind legs of the mice led to the significant contrast effect of T_1_-weighted MR images in the areas of interest (Figure 9c). To demonstrate the potential applications of DO3A-Gd-**CA** in biomedical diagnosis fields, the proposed probe was subcutaneously injected into one hind leg of mouse and then imaged at different time points, respectively. As shown in Figure S16, the successive bright MRI action of the DO3A-Gd-**CA** on mice model was achieved at four representative time points, even after 12 h, suggesting that the contrast agents could continuously improve contrast in tissues and have a relatively longer blood circulation time. The results of images demonstrated that DO3A-Gd-**CA** could serve as a MRI probe for imaging of fluoride ions in vivo.

## 4. Conclusions

In summary, we reported the design and synthesis of a novel mononuclear nine-coordinate complex, DO3A-Gd-**CA** as fluoride-specific fluorescence and MRI dual-modal probe. Upon the addition of fluoride ions to the aqueous solution of DO3A-Gd-**CA**, the replacement of the coordination aromatic carboxylic acid (**CA**) led an increase in longitudinal relaxivity (*r*_1_) and an enhancement in fluorescence intensity, realizing switch-on dual-modal responses towards fluoride ions in water medium. The desirable features of DO3A-Gd-**CA**, such as high sensitivity, suitability at physiological pH, favorable dynamical stability and low cytotoxicity encouraged us to further evaluate its potential biological applications. In vivo magnetic resonance imaging (MRI) in mice indicates that DO3A-Gd-**CA** can be potentially used as a powerful tool for the detection of fluoride ions in live systems. Given these promising results, we believe that DO3A-Gd-**CA** is valuable both in methodology and potential application, which provides a new flexible strategy for the rational design of bimodal or multimodal probes for anion sensing.

## Supplementary Materials

The following are available online at http://www.mdpi.com/1424-8220/16/12/2165/s1, Figures S1 and S2: NMR of **CA**, Figure S3–S5, S8, S11: HR MS characterizations, Figure S6: Benesi-Hildebrand plot, Figure S7: Stability of DO3A-Gd-**CA**, Figure S9: UV-Vis and emission spectra of **CA** sequential upon addition of [DO3A-Gd]^−^ and fluoride ion, Figure S10: Job’s plot, Figure S12: Linear relationship, Figure S13: Changes in the *r*_1_ of DO3A-Gd as a function of the **CA** concentration, Figure S14: Cell viabilities, Figure S15: Influence of pH on DO3A-Gd-**CA**, Figure S16: Resident time of DO3A-Gd-**CA** in mice.
