# Supplementary material for: Visualization of Fluoride Ions In Vivo Using a Gadolinium(III)-Coumarin Complex-Based Fluorescence/MRI Dual-Modal Probe
Source: Sensors (Basel). 2016 Dec 16;16(12):2165. doi: 10.3390/s16122165 (PMC5191144; doi:10.3390/s16122165)
Supplement: Supplementary file 1 [file sensors-16-02165-s001.pdf]

Supplementary Materials: Visualization of Fluoride Ion In Vivo Using a Gadolinium(III)-Coumarin Complex-Based Fluorescence/MRI Dual-Modal Probe

Yue Wang, Renfeng Song, Huan Feng, Ke Guo, Qingtao Meng, Haijun Chi, Run Zhang and Zhiqiang Zhang

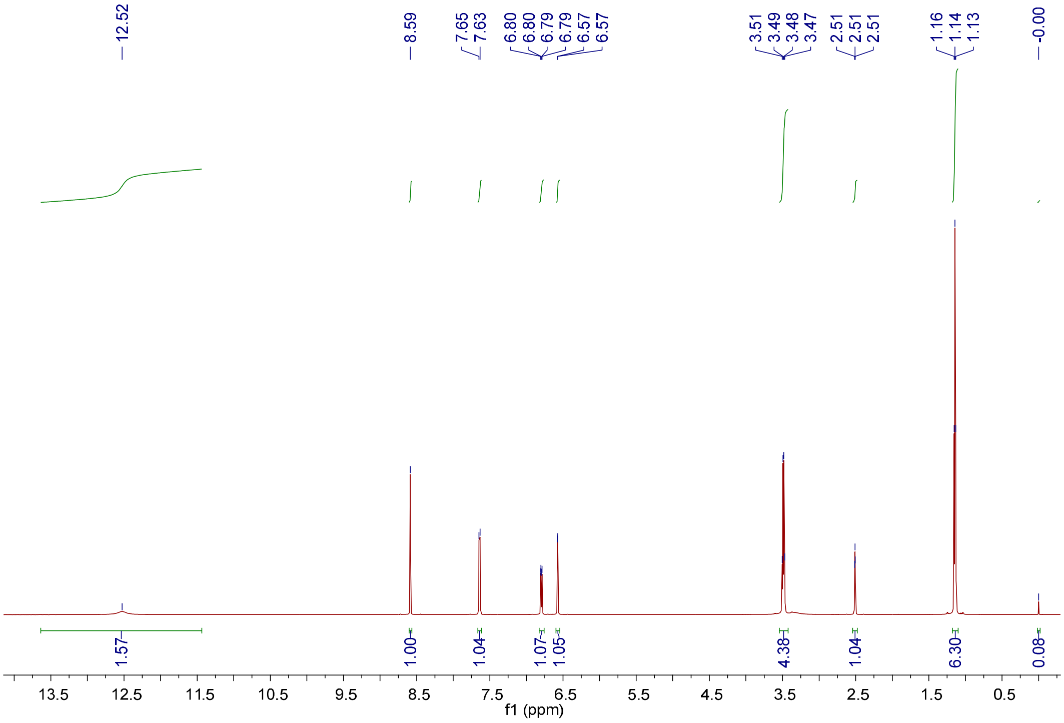

Figure S1. ^{1}H-NMR of **CA** (DMSO-*d*$_6$).

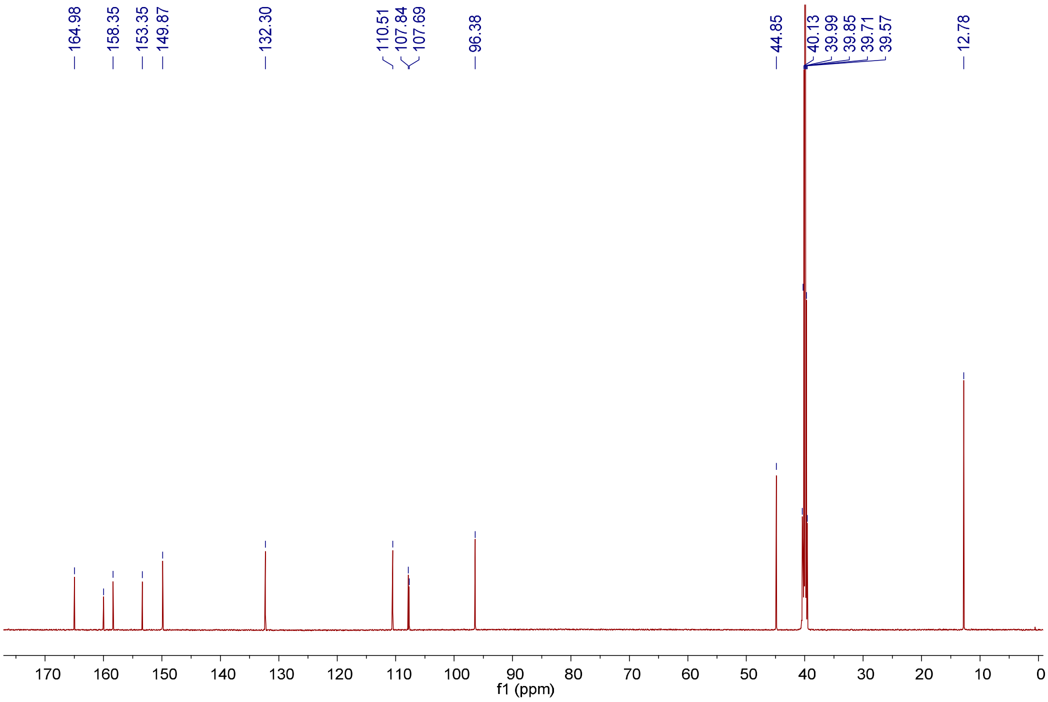

Figure S2. ^{13}C-NMR of **CA** (DMSO-*d*$_6$).

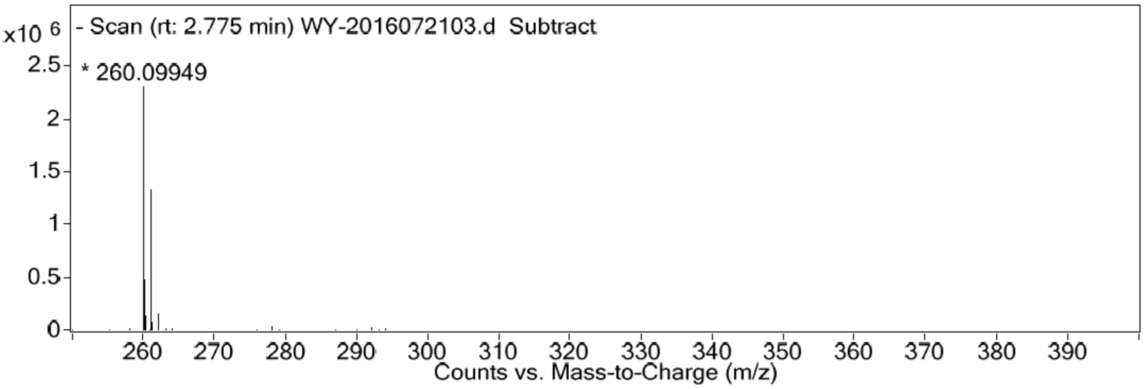

Figure S3. High resolution MS spectrum of 4-(diethylamino)-coumarin-3-carboxylic acid (**CA**).

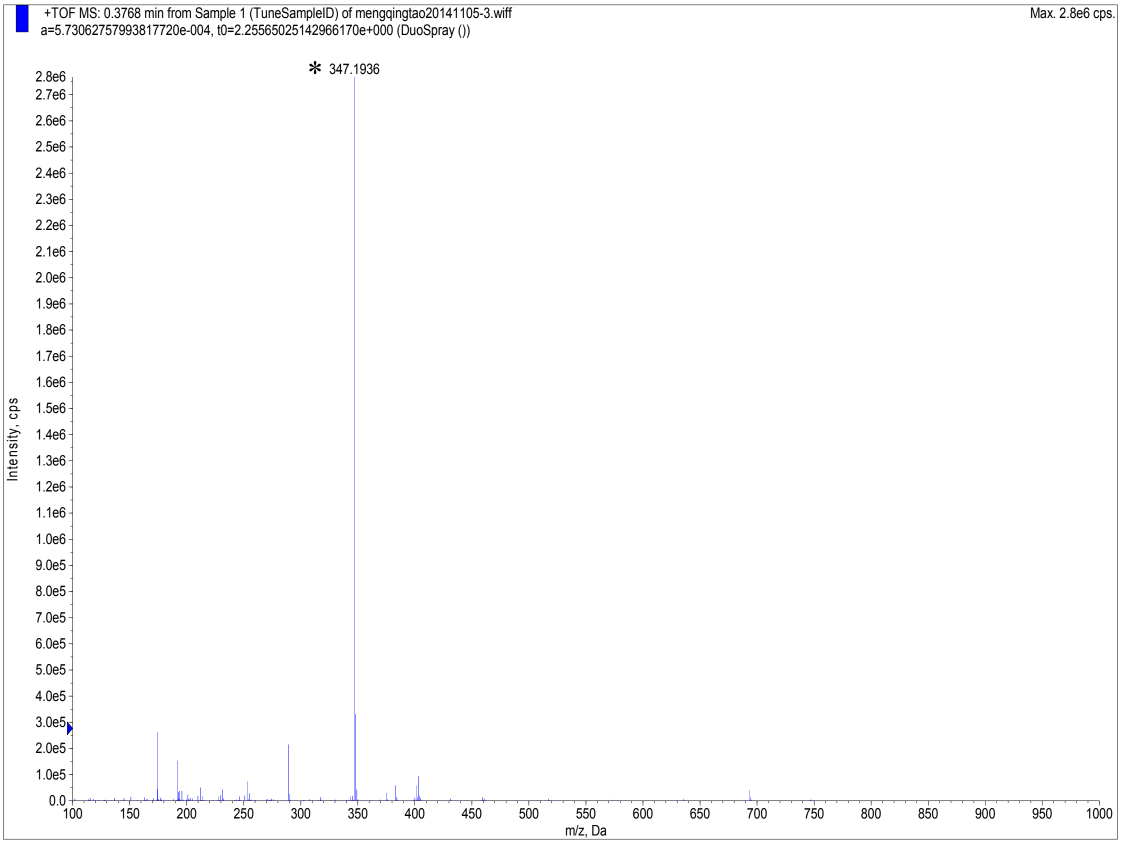

Figure S4. High resolution MS spectrum of 1,4,7-tris(carboxymethyl)-1,4,7,10-tetraazacyclododecane (**DO3A**).

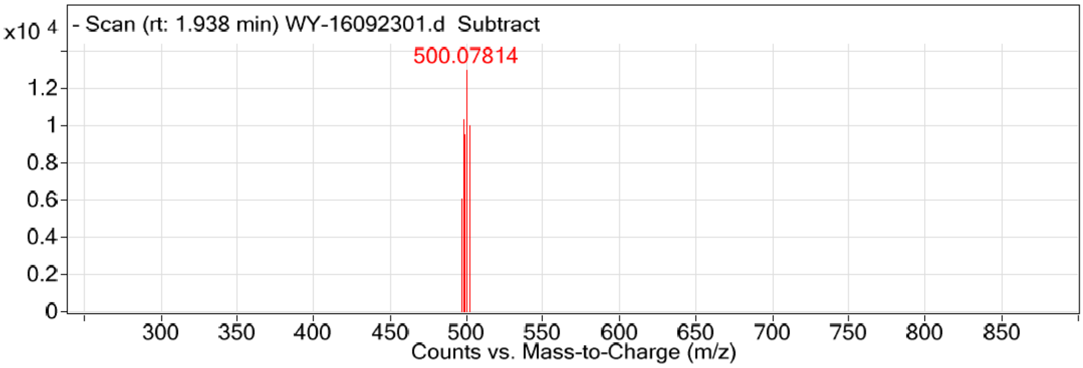

Figure S5. High resolution MS spectrum of [**DO3A**-Gd]$^{-}$.

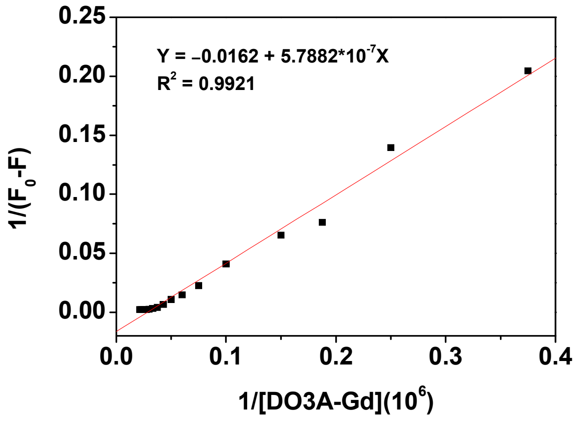

Figure S6. Benesi-Hildebrand plot (emission at 460 nm) of **CA** (10 μM) based on 1:1 binding stoichiometry with [DO3A-Gd]⁻. Excitation was performed at 408 nm.

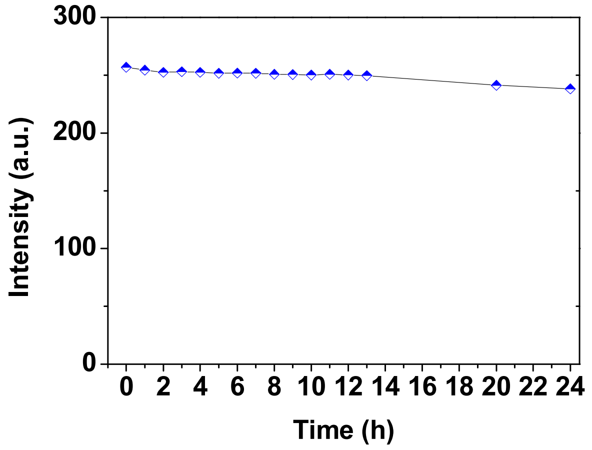

Figure S7. Fluorescence spectra of DO3A-Gd-**CA** (10 μM) at different times in CH_3CN-H_2O (9:1, *v*/*v*, pH = 7.4). The intensities were recorded at 460 nm, excitation at 408 nm.

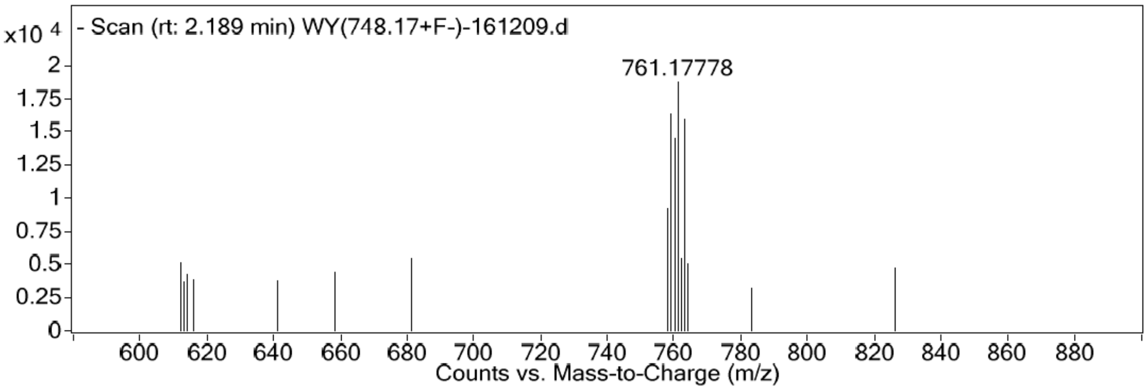

Figure S8. High resolution MS spectrum of [**DO3A**-Gd-**CA**]⁻.

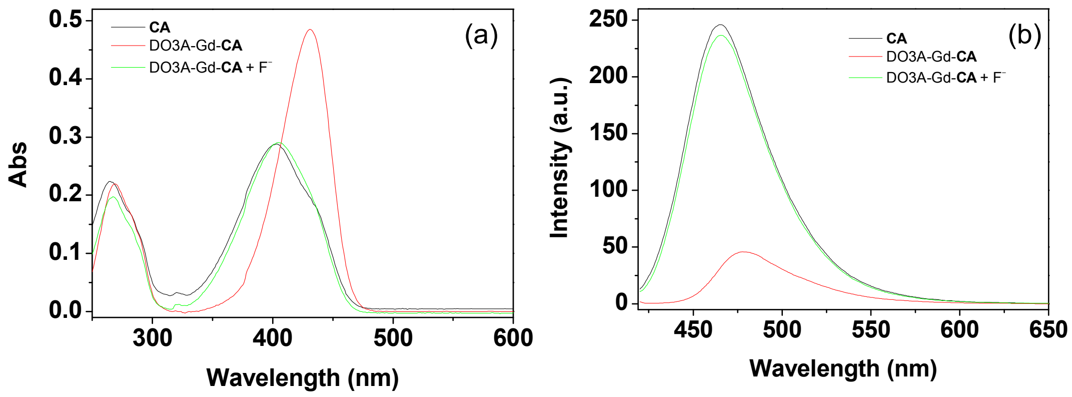

Figure S9. UV-vis absorption (**a**) and emission spectra (**b**) of **CA** (10 μM) sequential upon addition of [DO3A-Gd]Na (15 μM) and fluoride ion (200 μM) in aqueous (CH_3CN:H_2O = 9:1, pH = 7.4).

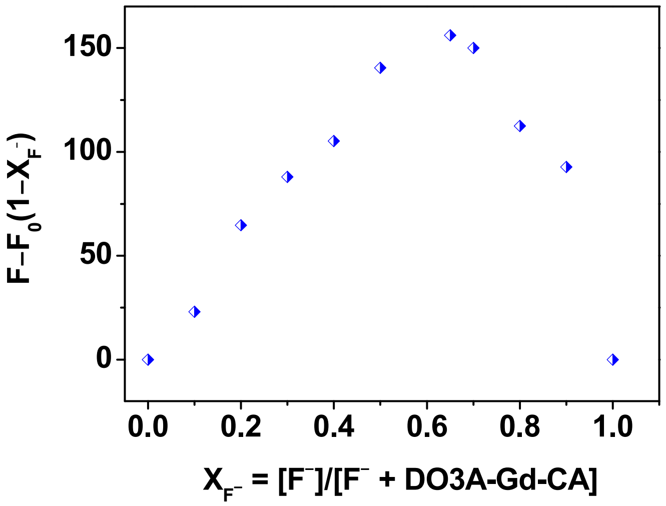

Figure S10. Job's plot of DO3A-Gd-**CA** toward fluoride ions in aqueous (CH_3CN:H_2O = 9:1, pH = 7.4).

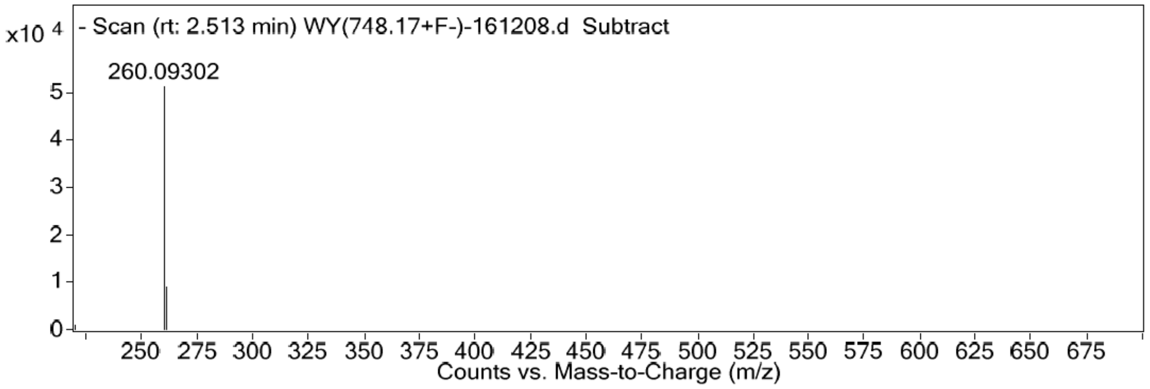

Figure S11. High resolution MS spectrum of DO3A-Gd-**CA** in presence of fluoride ions in aqueous.

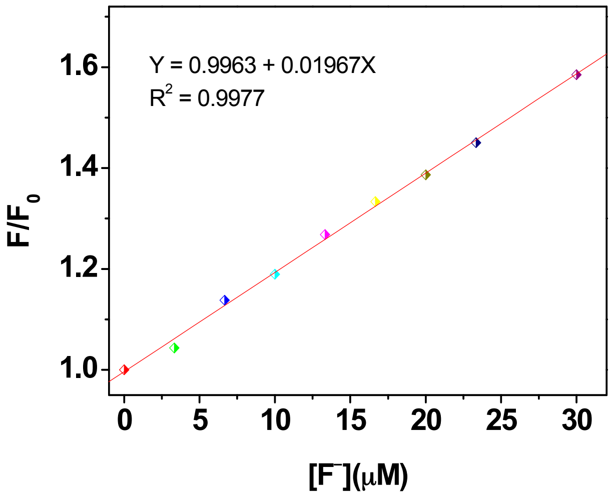

Figure S12. Linear relationship between fluorescence intensity of DO3A-Gd-**CA** (5 μM) at 460 nm versus the concentration of fluoride ion (0–30 μM) in aqueous (CH_3CN:H_2O = 9:1). Excitation was performed at 408 nm.

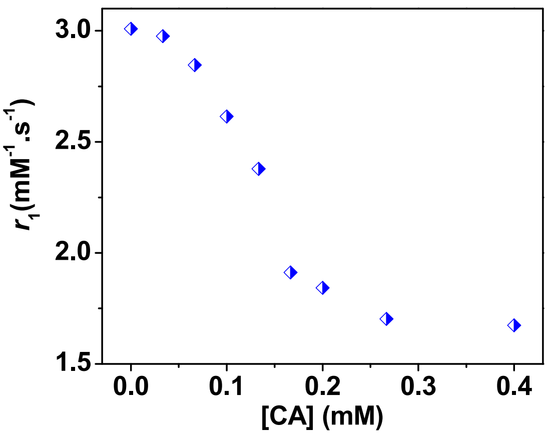

Figure S13. Changes in the longitudinal relaxivity (r_1) of DO3A-Gd (0.2 mM) as a function of the **CA** concentration (0–0.4 mM) in CH_3CN-H_2O (9:1, *v*/*v*, pH = 7.4).

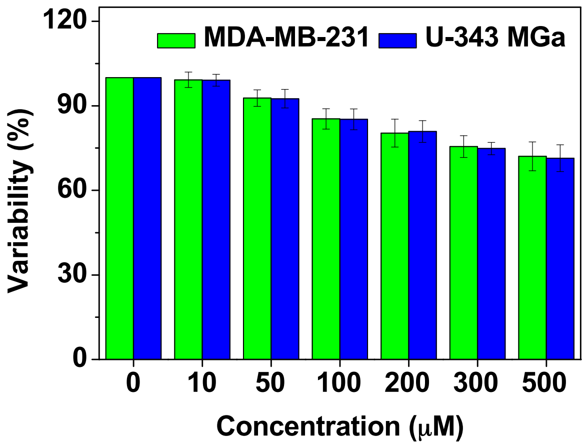

Figure S14. MDA-MB-231 and U-343 MGa cell viability values (%) assessed using an MTT proliferation test versus incubation concentrations of DO3A-Gd-**CA**.

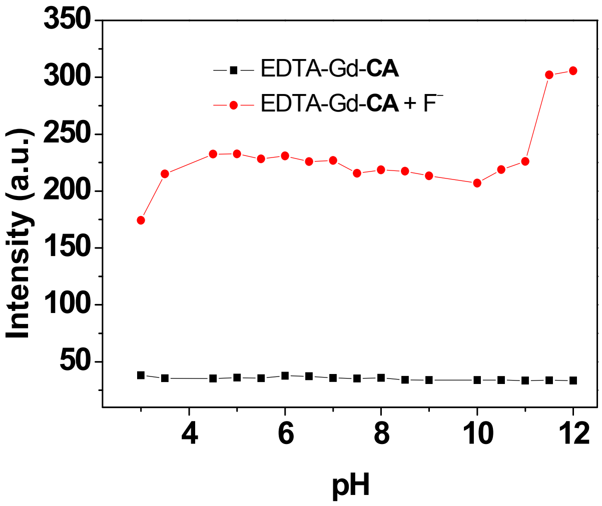

Figure S15. Influence of pH on the fluorescence intensities of DO3A-Gd-**CA** in the absence and presence of fluoride ion. The intensities were recorded at 460 nm, excitation was performed at 408 nm.

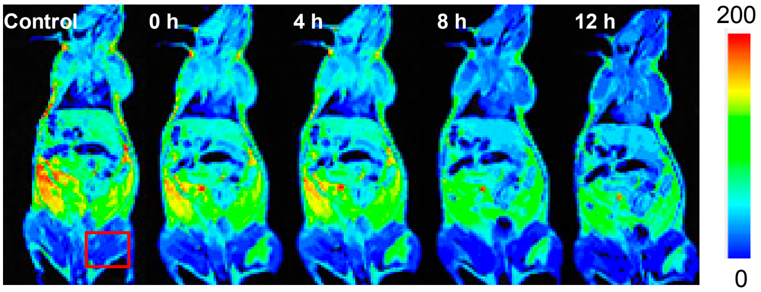

Figure S16. In vivo T_1-weighted MR pseudocolor images of mice at four representative time points after subcutaneous injection of DO3A-Gd-**CA** into one hind leg of mouse(0.2 mL, 0.2 mM).
